# Determinants of Longitudinal Change of Lung Function in Different Gender in a Large Taiwanese Population Follow-Up Study Categories: Original Investigation

**DOI:** 10.3390/jpm11101033

**Published:** 2021-10-15

**Authors:** Chia-Heng Chang, Szu-Chia Chen, Jiun-Hung Geng, Da-Wei Wu, Jiun-Chi Huang, Pei-Yu Wu

**Affiliations:** 1 Department of Post Baccalaureate Medicine, Kaohsiung Medical University, Kaohsiung 807, Taiwan; jerry81o72269@hotmail.com; 2 Department of Internal Medicine, Kaohsiung Municipal Siaogang Hospital, Kaohsiung Medical University, Kaohsiung 812, Taiwan; scarchenone@yahoo.com.tw (S.-C.C.); u8900030@yahoo.com.tw (D.-W.W.); karajan77@gmail.com (J.-C.H.); 3 Division of Nephrology, Department of Internal Medicine, Kaohsiung Medical University Hospital, Kaohsiung Medical University, Kaohsiung 807, Taiwan; 4 Faculty of Medicine, College of Medicine, Kaohsiung Medical University, Kaohsiung 807, Taiwan; 5 Research Center for Environmental Medicine, Kaohsiung Medical University, Kaohsiung 807, Taiwan; 6 Department of Urology, Kaohsiung Municipal Siaogang Hospital, Kaohsiung Medical University, Kaohsiung 812, Taiwan; u9001090@gmail.com; 7 Department of Urology, Kaohsiung Medical University Hospital, Kaohsiung Medical University, Kaohsiung 807, Taiwan; 8 Division of Pulmonary and Critical Care Medicine, Department of Internal Medicine, Kaohsiung Medical University Hospital, Kaohsiung Medical University, Kaohsiung 807, Taiwan

**Keywords:** lung function change, gender difference, follow-up, Taiwan biobank

## Abstract

Chronic lung disease is associated with tremendous social and economic burden worldwide. The aim of this study was to investigate the sex-specific risk factors for changes in lung function in a large longitudinal study. We included 9059 participants from the Taiwan Biobank. None of the participants had a history of smoking, asthma, emphysema or bronchitis. Lung function was assessed using spirometry measurements of forced vital capacity (FVC) and forced expiratory volume in 1 s (FEV1). Change in the FEV1/FVC (ΔFEV1/FVC) was calculated as a follow-up FEV1/FVC minus baseline FEV1/FVC. Linear regression analysis was used to identify associations between variables and ΔFEV1/FVC in the male and female participants. After multivariable adjustments, the male participants (vs. females; *p* = 0.021) were significantly associated with a low ΔFEV1/FVC. In addition, the male participants with low aspartate aminotransferase (AST) (*p* = 0.003), high alanine aminotransferase (ALT) (*p* = 0.006) and a low estimated glomerular filtration rate (eGFR) (*p* = 0.003) were significantly associated with a low ΔFEV1/FVC. For the female participants, low systolic blood pressure (*p* = 0.005), low diastolic blood pressure (*p* = 0.031), low AST (*p* < 0.001), high ALT (*p* < 0.001) and a low eGFR (*p* = 0.001) were significantly associated with a low ΔFEV1/FVC. In this large follow-up study, we found that the male participants had a faster decrease in the FEV1/FVC than the female participants. In addition, liver and renal functions were correlated with changes in lung function in both the male and female participants. Our findings provide useful information on sex-specific changes in lung function.

## 1. Introduction

Non-communicable disease has evolved, with growing sufferers and economic burden in modern society, and is characterized by high industrialization and an aging population [1]. Among the most notable pathologies, such as cardiovascular diseases, malignant neoplasms, diabetes, pulmonary and mental disorders, chronic respiratory disease was the third leading cause of death in 2017, behind cardiovascular diseases and neoplasms [2]. Facing these global public health challenges has demanded economic research, budget and policy making and population surveys, as well as many points of view that need to be tailored for each nation [1,3]. Additionally, research on the aging effect in humans becomes increasingly important as life expectancy increases. Several aspects have been explored with regard to the aging process of the respiratory system. At a molecular level, increased chemical cross-linking of collagen, which is a major component of the lungs, has been reported [4]. In addition, elastin, another major component of the lungs, has been shown to decrease in the alveolar walls [5]. These changes result in decreased lung compliance. At the macroscopic level, changes in the thoracic structure have been reported, including a loss of height and calcification of costal cartilages. The increase in rigidity and changes in shape contribute to a reduction in chest wall compliance [6]. In addition, a loss of respiratory muscle mass and reduction in muscle strength also contribute to a decline in respiratory function [7]. From the aspect of lung function, a decline in lung function has been observed in older adults [8].

Chronic obstructive pulmonary disease (COPD) has gained increasing attention over recent decades, and it was the third leading cause of death globally [9]. In Taiwan, the prevalence of COPD was 1194/100,000 in 2010, and it was the seventh leading cause of death [10]. COPD is associated with pain [11], weight loss [12], depression [13], cardiovascular disease [14], metabolic syndrome [15] and type 2 diabetes mellitus (DM) [16]. Important risk factors for COPD include genetic factors, exposure to tobacco smoke, air pollution, sex, infection and socioeconomic status [17]. A rapid decline in lung function has been associated with an increased risk of hospital admission and death [18], and COPD is associated with a rapid decline in lung function in adults [19]. Thus, it is important to investigate determinants that accelerate lung function decline.

A systematic review by Thomas et al. reported predictors associated with lung function decline including smoking, sex, body mass index (BMI), ethnicity and blood pressure [20]. In addition, Choi et al. also reported sex differences in pulmonary function [21]. Moreover, they found that blood pressure was independently associated with lung function in men, whereas blood pressure, fasting plasma glucose and high-density lipoprotein cholesterol were independently associated with lung function in women [21]. Due to the lack of large cohort follow-up studies on the association between sex and changes in lung function, the aims of this study were to investigate the risk factors and sex differences associated with changes in lung function in a longitudinal study of 9059 participants with no history of smoking, asthma, emphysema or bronchitis recruited from the Taiwan Biobank (TWB).

## 2. Materials and Methods

### 2.1. Ethics Statement

The Ethics and Governance Council of the TWB and the Institutional Review Board (IRB) on Biomedical Science Research, Academia Sinica, Taiwan granted ethical approval for the TWB. In addition, the IRB of Kaohsiung Medical University Hospital approved this study (KMUHIRB-E(I)-20190398), which was conducted according to the Declaration of Helsinki.

### 2.2. TWB

The TWB contains data on lifestyle and genomic factors of community-based Taiwanese volunteers aged 30 to 70 years with no history of cancer [22,23]. All of the participants signed informed consent forms, after which blood samples were obtained, and in-person interviews and physical examinations were conducted.

During the physical examinations, body height and weight were recorded, and the BMI was calculated (in kg/m^2^). Each participant completed a questionnaire during the in-person interviews with a TWB researcher. The questionnaire collected data on personal and family medical histories, lifestyle factors including regular exercise, diet and personal information. Regular exercise was defined as performing at least 30 min of physical activity, thrice weekly. Further, physical activity only referred to activities including playing a sport, computer-based dancing/exercise games/activities, cycling, hiking, swimming, yoga and jogging, etc. However, work-related physical activity or heavy manual work were not recorded.

**Figure 1:**
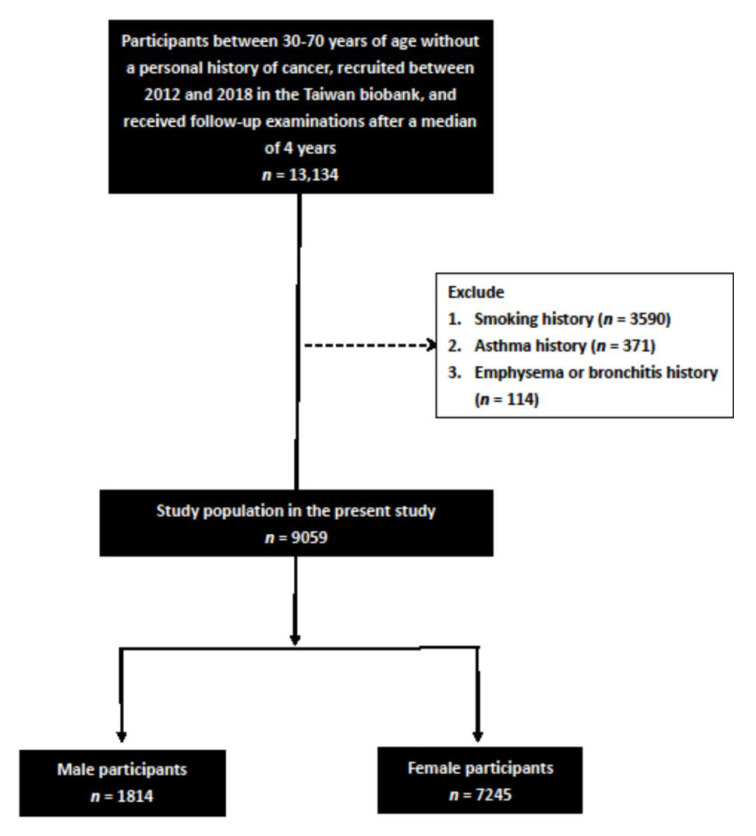
Flowchart of study population.

A total of 13,134 participants with a median of 4 years of complete spirometry data were identified in the TWB. We excluded participants who had a history of smoking (*n* = 3590), asthma (*n* = 371) and emphysema or bronchitis (*n* = 114). The remaining 9059 participants (1814 males and 7245 females, mean age 51.0 ± 10.2 years) were included in this study (Figure 1).

### 2.3. Laboratory, Medical Demographic Data

The following variables were recorded at baseline: age, sex, histories of DM and hypertension, estimated glomerular filtration rate (eGFR), systolic blood pressure (SBP), diastolic blood pressure (DBP), alanine aminotransferase (ALT), aspartate aminotransferase (AST), fasting glucose, total cholesterol, triglycerides, hemoglobin and uric acid. The eGFR was calculated using the 4-variable Modification of Diet in Renal Disease equation [24].

### 2.4. Spirometry Measurements

Forced expiratory volume in 1 s (FEV1) and forced vital capacity (FVC) were measured (in L) using a Micro Labs spirometer and Spida 5 software (Micro Medical Ltd., Rochester, Kent, UK). All measurements were made by a trained technician following the 2005 European Respiratory Society and American Thoracic Society technical standards (i.e., differences within 5% or 100 mL) [25]. Each participant underwent three lung function tests and the best result was used in the analysis. Reference values were calculated using formulae based on a general Asian population and age, sex and height. Predicted FVC (or predicted FVC%) and predicted FEV1 (or predicted FEV1%) were calculated as the measurements divided by the reference values. Spirometry software was used to obtain predicted FVC% and predicted FEV1%. The change in the FEV1/FVC (ΔFEV1/FVC) was calculated as the follow-up FEV1/FVC minus baseline FEV1/FVC.

### 2.5. Statistical Analysis

Data are presented as a percentage or mean ± standard deviation. A chi-square and independent t tests were used to compare differences between groups for categorical and continuous variables, respectively. Linear regression analysis was used to identify associations between variables and ΔFEV1/FVC. A *p* value of <0.05 was considered to indicate a statistically significant difference. SPSS version 20.0 for Windows (SPSS Inc., Chicago, IL, USA) was used for all statistical analyses.

## 3. Results

### 3.1. Comparisons of the Clinical Characteristics between the Male and Female Participants

Comparisons of the clinical characteristics between the male and female groups are shown in Table 1. Compared to the male participants, the female participants had a lower prevalence of DM, lower prevalence of hypertension, less regular exercise, lower SBP, lower DBP, lower BMI, lower fasting glucose, lower hemoglobin, lower triglycerides, higher total cholesterol, lower AST, lower ALT, higher eGFR and lower uric acid. Regarding lung function, compared to the male participants, the female participants had a lower FVC at baseline, lower FVC at follow-up, lower FEV1 at baseline, lower FEV1 at follow-up, lower FEV1/FVC at baseline and a higher FEV1/FVC at follow-up.

### 3.2. Determinants of ΔFEV1/FVC Using Multivariable Linear Regression Analysis

The determinants of ΔFEV1/FVC were examined using multivariable linear regression analysis, which revealed that the participants who were older (per 1 year; coefficient β, −0.055; *p* = 0.041), male (vs. female; coefficient β, −1.602; *p* = 0.021), had a low SBP (per 1 mmHg; coefficient β, 0.071; *p* = 0.001), low DBP (per 1 mmHg; coefficient β, 0.084; *p* = 0.011), high BMI (per 1 kg/m^2^; coefficient β, −0.175; *p* = 0.015), low AST (per 1 U/L; coefficient β, 0.314; *p* < 0.001), high ALT (per 1 U/L; coefficient β, −0.196; *p* < 0.001) and a low eGFR (per 1 mL/min/1.73 m^2^; coefficient β, 0.039; *p* < 0.001), which were significantly associated with a low ΔFEV1/FVC (Table 2).

### 3.3. Determinants of ΔFEV1/FVC in the Male and Female Participants Using Multivariable Linear Regression Analysis

The determinants of the ΔFEV1/FVC in the male and female participants were examined using multivariable linear regression analysis (Table 3). The results show that the male participants with a low AST (per 1 U/L; coefficient β, 0.226; *p* = 0.003), high ALT (per 1 U/L; coefficient β, −0.131; *p* = 0.006) and a low eGFR (per 1 mL/min/1.73 m^2^; coefficient β, 0.080; *p* = 0.003) were significantly associated with a low ΔFEV1/FVC. In comparison, the female participants with a low SBP (per 1 mmHg; coefficient β, 0.067; *p* = 0.005), low DBP (per 1 mmHg; coefficient β, 0.081; *p* = 0.031), low AST (per 1 U/L; coefficient β, 0.345; *p* < 0.001), high ALT (per 1 U/L; coefficient β, −0.222; *p* < 0.001) and a low eGFR (per 1 mL/min/1.73 m^2^; coefficient β, 0.033; *p* = 0.001) were significantly associated with a low ΔFEV1/FVC.

However, the interactions of all variables and sex did not achieve significance.

## 4. Discussion

In this longitudinal analysis, we investigated the risk factors and sex-specific differences in changes in lung function in a longitudinal study of 9059 Taiwanese participants who were followed up for a median of 4 years. We found that the male participants had a faster decrease in FEV1/FVC than the female participants. Further, a low AST, high ALT and low eGFR in the male participants, and a low SBP, low DBP, low AST, high ALT and low eGFR in the female participants were associated with a rapid decrease in FEV1/FVC.

The first important finding of this study is that after excluding a history of smoking, asthma, emphysema and bronchitis, the male participants had a faster decrease in FEV1/FVC after follow-up than the female participants. Thomas et al. conducted a systematic review [20] investigating age-related changes in lung function in order to avoid overdiagnosing healthy individuals. The review included 16 cohort studies with 31,099 participants without known lung diseases, and all of the studies demonstrated a decline in lung function with age. With regards to sex differences, the authors found a faster decline in FEV1 and FVC in males than in females [20]. In addition, Sharma and Goodwin [7] reviewed anatomical, physiological and immunological changes in the respiratory system with age, and found sex differences in the decline in lung function with regards to respiratory muscle function. Moreover, Enright et al. reported a larger decrease in maximal inspiratory pressure, which represents the strength of the diaphragm, in men than in women, between the ages of 65 and 85 years [26]. Besides the respiratory system itself, sex-related differences in other systems may also play important roles in lung function decline. For example, in a 20-year longitudinal study conducted in Europe [27], Triebner et al. investigated 275 women who had received hormone replacement therapy and 383 nonusers, and demonstrated that women who had used hormone replacement therapy for more than 5 years had a slower decline in lung function than the nonusers. Straub [28] also reported that estrogens had an anti-inflammatory effect on aging lungs. These findings suggest that endogenous sex hormones may protect the lungs against aging.

In this study, we also found that a high BMI was associated with a rapid decrease in FEV1/FVC. Many studies have reported an association between obesity and lung function, and that BMI can affect the rate of lung function decline in healthy individuals [20]. Bartholomew et al. and Triebner et al. [29,30] reported that a higher BMI was associated with a lower FEV1 and FVC, and that this could be explained by inflammatory adipose tissue. In a cross-sectional study conducted in Korea, Kim et al. [31] also found that a higher BMI was associated with a lower FVC in participants both with and without metabolic syndrome. However, they found a positive correlation between FVC and BMI in the subgroup with a BMI < 25 kg/m^2^, which they attributed to an increase in muscle mass. Nevertheless, fat distribution has been shown to be a more important factor than BMI when evaluating the impact of obesity on pulmonary function [32]. In addition, higher expressions of proinflammatory cytokines such as interleukin-1β, tumor necrosis factor-α, interleukin-6, interleukin-8, monocyte chemoattractant protein-1 and adipokine leptin have been demonstrated in obese individuals, and they could potentially be predictors of lung function [33]. Moreover, van Huistede et al. found that relative eosinophilia in morbidly obese participants was significantly associated with a lower FEV1/FVC ratio [34]. In general, the influence of obesity on lung function can be through both mechanical and inflammatory effects.

Another important finding of this study is that a low SBP and DBP were associated with a rapid decrease in FEV1/FVC in the female participants. Many studies have reported an association between hypertension and a lower FEV1 or FVC, though no difference in FEV1/FVC due to the proportional reduction in FEV1 and FVC [35,36,37]. Koo et al. reported that participants with hypertension had faster annual rates of decline in FVC than those without hypertension [38]. Arterial stiffness and microangiopathy caused by hypertension may contribute to the association [38]. Elevated blood pressure can cause accelerated stiffening of both central arteries and pulmonary vasculature, and decreased pulmonary vascular elasticity would most likely affect pulmonary function, regardless of any parenchymal changes [39]. However, in this study, we found that a low SBP and DBP contributed to a rapid decline in lung function. A previous report also demonstrated an association between a low DBP and low FEV1/FVC [40], and another study reported that orthostatic hypotension had an impact on lung circulation, especially at the apices of the lungs [41]. We hypothesize that chronic low blood pressure may result in hypoxemia, due to ventilation/perfusion mismatch. Hypoxemia has been reported to induce oxidative stress and inflammatory cytokines, causing injury to the lung parenchyma [42].

In this study, we also found that a low AST and high ALT were associated with a rapid decrease in FEV1/FVC in both the male and female participants. Interestingly, the impact of AST and ALT on lung function decline in our study seems to be inverse. Previous studies have shown that nonalcoholic fatty liver disease (NAFLD) is associated with the inflammatory status of the liver, and that this can lead to a cascade that affects the lungs over the long term [43,44]. The concept of a low-grade inflammation effect is similar to obesity. Furthermore, a chronic inflammatory condition usually results from multiple origins. Jung et al. demonstrated that both FVC and FEV1 were inversely associated with the presence of NAFLD, after adjusting for the BMI and the metabolic syndrome [43]. In a study conducted in Romania [45], Rascu et al. studied liver function in a small asthmatic population without hepatic diseases, and found that a lower AST and ALT were associated with a lower FEV1 and FEV1/FVC. They speculated that a lower AST and ALT could be caused by a higher degree of sarcopenia. This highlights that, to some extent, lower aminotransferase levels represent abnormal status in various diseases, including liver cirrhosis, uremia, malnutrition and DM [46]. In addition, AST and ALT originate from multiple organs and represent various pathophysiological statuses, which makes it difficult to interpret.

Another important finding of this study is that a low eGFR was significantly associated with a fast decrease in FEV1/FVC in both the male and female participants. Lim et al. reported that glomerular hyperfiltration was associated with a restrictive pattern of spirometry after adjusting for blood pressure and metabolic indices, but that confounders, such as reduced muscle mass, low serum creatinine and an inflammatory effect still existed [47]. In addition, Kim et al. [40] performed an analysis of the Korean National Health and Nutrition Examination Survey, which represents the general Korean population from 2010 to 2012, and found that both restrictive and obstructive impaired pulmonary function was associated with a decline in the eGFR in both male and female participants. Sankar et al. conducted a National Health and Nutrition Examination Survey (NHANES)-based retrospective study, and reported a statistical correlation between lung function and renal function [42]. The renal and pulmonary function of the participants in the NHANES varied with regard to spirometry, eGFR and urine albumin to creatinine ratio. The authors found that a lower eGFR was associated with higher odds of having obstructive lung function but lower odds of having restrictive lung function. In addition to the eGFR, the authors also found that albuminuria was independently associated with higher odds of both obstructive and restrictive lung function [42]. Moreover, they proposed that inflammation triggered both patterns of lung function decline, which resulted in protein loss into urine. Both albuminuria and a decline in the eGFR indicate kidney damage.

The main strengths of this study include the large number of participants and comprehensive follow-up data. However, several limitations should also be noted. First, the TWB does not contain data on medications, so we could not examine the effect of medications on changes in lung function. In addition, approximately 50% of the participants in the TWB returned for follow-up examinations, which may have resulted in sample bias and in turn affected the interpretation of our results. Finally, all participants in the TWB are of Chinese ethnicity, and as such our findings may not be generalizable to other populations.

In conclusions, in this large follow-up study, we found that the male participants had a faster decline in FEV1/FVC than the female participants. In addition, liver and renal functions were correlated with changes in lung function in both the male and female participants. Our findings provide useful information on sex-specific changes in lung function.

## Figures and Tables

**Table 1:** Comparison of clinical characteristics among participants according to different gender.

Characteristics	Male (*n* = 1814)	Female (*n* = 7245)	*p*
Age (year)	50.6 ± 11.6	51.1 ± 9.9	0.067
DM (%)	5.5	4.0	0.004
Hypertension (%)	15.2	10.3	<0.001
Regular exercise habits (%)	51.9	48.8	0.019
Menopause (%)		44.3	
SBP (mmHg)	121.5 ± 16.1	114.6 ± 17.8	<0.001
DBP (mmHg)	76.6 ± 10.2	69.8 ± 10.3	<0.001
BMI (kg/m^2^)	24.8 ± 3.2	23.4 ± 3.4	<0.001
Laboratory parameters			
Fasting glucose (mg/dL)	98.1 ± 19.0	93.9 ± 17.0	<0.001
Hemoglobin (g/dL)	15.0 ± 1.1	13.0 ± 1.3	<0.001
Triglyceride (mg/dL)	121.2 ± 88.5	102.2 ± 67.7	<0.001
Total cholesterol (mg/dL)	191.0 ± 34.4	197.5 ± 35.3	<0.001
AST (U/L)	25.9 ± 10.6	23.7 ± 10.4	<0.001
ALT (U/L)	27.5 ± 17.8	20.9 ± 15.9	<0.001
eGFR (mL/min/1.73 m^2^)	98.8 ± 19.7	114.0 ± 25.6	<0.001
Uric acid (mg/dL)	6.4 ± 1.3	4.9 ± 1.1	<0.001
Lung function			
FVC (L, baseline)	3.6 ± 0.7	2.4 ± 0.5	<0.001
FVC (L, follow-up)	3.3 ± 0.7	2.2 ± 0.5	<0.001
FEV1 (L, baseline)	2.6 ± 0.8	1.8 ± 0.6	<0.001
FEV1 (L, follow-up)	2.9 ± 0.7	2.0 ± 0.5	<0.001
FEV1/FVC (%, baseline)	73.7 ± 18.4	72.5 ± 18.4	0.017
FEV1/FVC (%, follow-up)	86.9 ± 10.2	87.5 ± 10.7	0.037

Abbreviations. DM, diabetes mellitus; SBP, systolic blood pressure; DBP, diastolic blood pressure; BMI, body mass index; AST, aspartate aminotransferase; ALT, alanine aminotransferase; eGFR, estimated glomerular filtration rate; FVC, forced vital capacity; FEV1, forced expiratory volume in 1 s.

**Table 2:** Determinants for △FEV1/FVC using multivariable linear regression analysis.

Characteristics	Multivariable	
	Unstandardized Coefficient β (95% CI)	*p*
Age (per 1 year)	−0.055 (−0.108, −0.002)	0.041
Male (vs. female)	−1.602 (−2.966, −0.239)	0.021
DM	0.029 (−2.330, 2.387)	0.981
Hypertension	−0.566 (−2.025, 0.893)	0.447
Regular exercise habits	−0.530 (−1.421, 0.361)	0.243
SBP (per 1 mmHg)	0.071 (0.029, 0.112)	0.001
DBP (per 1 mmHg)	0.084 (0.019, 0.148)	0.011
BMI (per 1 kg/m^2^)	−0.175 (−0.317, −0.034)	0.015
Laboratory parameters		
Fasting glucose (per 1 mg/dL)	−0.002 (−0.030, 0.027)	0.910
Hemoglobin (per 1 g/dL)	−0.025 (−0.386, 0.337)	0.894
Triglyceride (per 1 mg/dL)	−0.002 (−0.009, 0.004)	0.442
Total cholesterol (per 1 mg/dL)	0.012 (−0.001, 0.025)	0.077
AST (per 1 U/L)	0.314 (0.257, 0.390)	<0.001
ALT (per 1 U/L)	−0.196 (−0.246, −0.146)	<0.001
eGFR (per 1 mL/min/1.73 m^2^)	0.039 (0.020, 0.057)	<0.001
Uric acid (per 1 mg/dL)	0.274 (−0.142, 0.690)	0.196

Values expressed as unstandardized coefficient β and 95% confidence interval (CI). Abbreviations are the same as in Table 1.

**Table 3:** Determinants for △FEV1/FVC in different genders using multivariable linear regression analysis.

Characteristics	Male (*n* = 1814)		Female (*n* = 7245)		
	Multivariable		Multivariable		
	Unstandardized Coefficient β (95% CI)	*P*	Unstandardized Coefficient β (95% CI)	*P*	Interaction *p*
Age (per 1 year)	−0.089 (−0.192, 0.014)	0.090	−0.032 (−0.119, 0.055)	0.472	0.138
DM	3.844 (−1.030, 8.718)	0.122	−0.955 (−3.660, 1.749)	0.489	0.281
Hypertension	−0.543 (−3.369, 2.284)	0.706	−0.439 (−2.148, 1.271)	0.615	0.733
Regular exercise habits	0.023 (−1.939, 1.984)	0.982	−0.695 (−1.696, 0.307)	0.174	0.919
Menopause	−	−	−0.052 (−1.621, 1.516)	0.948	−
SBP (per 1 mmHg)	0.080 (−0.012, 0.172)	0.090	0.067 (0.020, 0.114)	0.005	0.767
DBP (per 1 mmHg)	0.101 (−0.035, 0.238)	0.146	0.081 (0.008, 0.154)	0.031	0.916
BMI (per 1 kg/m^2^)	−0.252 (−0.580, 0.076)	0.132	−0.151 (−0.310, 0.007)	0.061	0.716
Laboratory parameters					
Fasting glucose (per 1 mg/dL)	−0.033 (0.092, 0.026)	0.270	0.007 (−0.025, 0.039)	0.682	0.516
Hemoglobin (per 1 g/dL)	−0.322 (−1.236, 0.592)	0.490	0.009 (−0.394, 0.412)	0.966	0.404
Triglyceride (per 1 mg/dL)	−0.002 (−0.013, 0.009)	0.729	−0.003 (−0.010, 0.005)	0.515	0.894
Total cholesterol (per 1 mg/dL)	0.014 (−0.015, 0.042)	0.337	0.010 (−0.005, 0.025)	0.182	0.626
AST (per 1 U/L)	0.226 (0.078, 0.373)	0.003	0.345 (0.255, 0.435)	<0.001	0.770
ALT (per 1 U/L)	−0.131 (−0.225, −0.037)	0.006	−0.222 (−0.282, −0.162)	<0.001	0.958
eGFR (per 1 mL/min/1.73 m^2^)	0.080 (0.028, 0.133)	0.003	0.033 (0.013, 0.053)	0.001	0.150
Uric acid (per 1 mg/dL)	0.708 (−0.078, 1.495)	0.078	0.113 (−0.383, 0.610)	0.655	0.341

Values expressed as unstandardized coefficient β and 95% confidence interval (CI). Abbreviations are the same as in Table 1.

## Data Availability

The data underlying this study is from the Taiwan Biobank. Due to restrictions placed on the data by the Personal Information Protection Act of Taiwan, the minimal data set cannot be made publicly available. Data may be available upon request to interested researchers. Please send data requests to: Szu-Chia Chen, PhD, MD. Division of Nephrology, Department of Internal Medicine, Kaohsiung Medical University Hospital, Kaohsiung Medical University.
